# Supplementary material for: A Reliability Generalization Meta-Analysis of the Antisocial Process Screening Device
Source: Behav Sci (Basel). 2025 Jun 25;15(7):860. doi: 10.3390/bs15070860 (PMC12292449; doi:10.3390/bs15070860)
Supplement: Supplementary file 1 [file behavsci-15-00860-s001.zip › behavsci-3572071-supplementary.pdf]

Supplemental Table 1

Model Specification of the Frick et al. (2000) Three-Factor Solution of the APSD

Items	Description	Ratings
Callous-Unemotional		
3	Concerned about schoolwork (R)	0 1 2
7	Keeps promises (R)	0 1 2
12	Feels bad or guilty (R)	0 1 2
18	Concerned about the feelings of others (R)	0 1 2
19	Does not show emotions	0 1 2
20	Keeps the same friends (R)	0 1 2
Narcissism		
5	Shallow emotions	0 1 2
8	Brags about accomplishments	0 1 2
10	Uses or cons others	0 1 2
11	Teases other people	0 1 2
14	Charming in insincere ways	0 1 2
15	Becomes angry when corrected	0 1 2
16	Thinks s/he is better or more important	0 1 2
Impulsivity		0 1 2
1	Blames others for their mistakes	0 1 2
4	Acts without thinking	0 1 2
9	Gets bored easily	0 1 2
13	Engages in risky and dangerous behavior	0 1 2
17	Does not plan ahead	0 1 2
Unloaded Items		
2	Engages in illegal activities	0 1 2
6	Lies easily and skillfully	0 1 2

Notes. (R) = Reversed scored items.

Supplemental Table 2

Information Extracted From Studies Included in Meta-Analysis

Author	M_{age}	SD_{age}	Country	Male	*N*	Language	Version	Sample	*SD*	*M*	α	Items
**Callous-Unemotional**												
Pijper et al. (2018)	10.350	1.270	Netherlands	100.00	49	Dutch	Mixed	Clinical	2.250	6.860	0.560	6
Romero et al. (2018)	7.320	2.690	Spain	48.60	449	Spanish	TR	Community			0.730	6
Bansal et al. (2018)b	9.600	1.700	Canada	73.13	62		TR	Mixed	2.970	4.850	0.710	6
Bansal et al. (2018)a	9.600	1.700	Canada	73.13	67		PR	Mixed	2.530	4.390	0.720	6
Gao et al. (2018)a	11.350	2.610	China	63.50	301	Chinese	PR	Community			0.440	6
Gao et al. (2018)b	11.350	2.610	China	63.50	301	Chinese	SR	Community			0.480	6
Oberth et al. (2017)			USA	58.00	753	English	PR	Community			0.650	6
Maurer et al. (2017)	17.380	0.860	USA	100.00	97	English	SR	Detained	1.690	5.620	0.430	6
Thomson & Centifant (2017)	9.900	0.710	UK	54.55	110	English	TR	Clinical			0.840	6
Wright et al. (2017)	2.570	2.310	UK	51.03	241	English	PR	Community			0.560	6
Colins et al. (2017)	16.250	1.070	Belgium	0.00	95		SR	Detained	1.880	4.210	0.310	6
Pechorro et al. (2017f)	16.230	1.380	Portugal	0.00	377	Portuguese	SR	Mixed			0.510	6
Pechorro et al. (2017d)	16.750	1.410	Portugal	100.00	221	Portuguese	SR	Detained			0.680	6
Haas et al. (2017)	10.500	1.300	USA	45.00	124	English	TR	Community			0.770	6
Ooi et al. (2017)b	10.60	1.91	Singapore	87.60	282		PR	Clinical	1.800	6.350	0.38	6
Ooi et al. (2017)a	10.60	1.91	Singapore	87.60	282		SR	Clinical	2.260	5.870	0.41	6
Zhang et al. (2017)	8.600	1.650	China	65.09	579	Chinese	PR	Clinical			0.700	6
Robinson et al. (2016)	8.800	0.970	USA	100.00	75	English	Mother	Community	2.130	3.920	0.640	6
Shaffer et al. (2016)	13.070	0.390	Canada	44.48	335		SR	Community	1.740	2.950	0.440	6
Masi et al. (2016)	9.60	1.33	Italy	90.00	144		PR	Clinical	2	5.67	0.75	6
Colins et al. (2016)a	16.220	1.130	Belgium	0.00	75		SR	Community	1.760	4.210	0.260	6
Colins et al. (2016)b	16.220	1.130	Belgium	0.00	75		PR	Community	3.030	7.280	0.640	6
Muratori et al. (2016)			Italy	89.83	59	Italian	PR	Clinical	1.830	5.890	0.770	6
Kahn et al. (2016)	17.030	1.100	USA	83.70	141	English	SR	Detained	1.770	4.860	0.530	6
O'Connor et al. (2016)	12.600		England	68.00	271	English	PR	Clinical	2.460	4.260	0.750	6
Zheng et al. (2016)	6.540	0.580	USA	58.00	753	English	Mixed	Mixed	0.370	0.630	0.650	6
Goodwin et al. (2015)	15.160	1.610	USA	68.07	451	English	SR	Community			0.500	7
Pasalich et al. (2015)	6.500	0.480	USA	69.00	891	English	PR	Community			0.640	6
Jezior et al. (2015)b	7.340	1.090	USA	69.10	184		PR	Community	1.940	2.910	0.600	6
Jezior et al. (2015)a	7.340	1.090	USA	69.10	141	English	TR	Community	2.460	4.260	0.690	6
Drislane et al. (2015)	18.800	1.630	USA	44.40	618	English	SR	Community			0.510	6
Pechorro et al. (2015c)	16.750	1.410	Portugal	100.00	221	Portuguese	SR	Detained			0.680	6
Muratori et al. (2015)	10.350	1.710	Italy	87.00	98	Italian	PR	Clinical	1.960	5.210	0.730	6
Oshukova et al. (2015)	15.060	0.280	Finland	46.80	372	Finnish	SR	Community	0.310	0.630	0.330	6
Childs et al. (2014)a	10.560	0.560	USA	59.60	120	English	Mixed	Community	0.380	1.030	0.710	6
Sharp & Vanwoerden (2014)	15.390	1.450	USA	38.50	346	English	SR	Clinical	2.100	3.760	0.530	6
Shenk et al. (2014)	8.980	1.710	USA	100.00	66	English	TR	Clinical			0.790	6
Ficks et al. (2014)a	10.580	3.200	USA	100.00	867	English	PR	Community	4.120	7.900	0.530	6

Ficks et al. (2014)b	10.570	3.180	USA	0.00	903	English	PR	Community	4.230	7.300	0.540	6
Pasalich et al. (2014)	9.580	1.600	Canada	75.70	111	English	Mixed	Mixed			0.760	6
Warren et al. (2014)	9.740	0.750	UK	79.63	54	English	TR	Clinical			0.600	6
McKenzie & Lee (2014)a	7.860	1.150	USA		221	English	PR	Mixed			0.600	6
McKenzie & Lee (2014)b	7.860	1.150	USA		153	English	TR	Mixed			0.700	6
Wied et al. (2014)	16.220	0.520	Netherlands	49.00	307	English	Mixed	Community			0.540	6
Silva et al. (2014)	16.500	1.280	Spain	90.38	104	Spanish	SR	Detained	2.520	6.720	0.700	6
Högström et al. (2013)	6.650	2.290	Sweden	54.39	57	Swedish	PR	Clinical			0.620	6
Dong et al. (2013)a	10.570	3.190	USA	49.00	1,681		PR	Community	3.640	9.380	0.610	6
Dong et al. (2013)b	10.820	3.390	USA	70.00	251		PR	Clinical	4.240	10.020	0.620	6
Joseph et al. (2013)a	14.010	1.820	UK	47.30	112	English	Mixed	Mixed			0.660	6
Joseph et al. (2013)b	14.010	1.820	UK	47.30	112	English	TR	Mixed			0.810	6
Kahn et al. (2013)a	13.430	1.860	USA	51.00	272	English	SR	Clinical	1.93	4.52	0.400	6
Kahn et al. (2013)b	13.430	1.860	USA	51.00	272	English	PR	Clinical	2.07	5.62	0.590	6
Brammer et al. (2012)	7.400	1.100	USA	69.10	230	English	Mixed	Community			0.590	6
Falk & Lee (2012)	7.400	1.100	USA	71.15	208	English	PR	Community			0.600	6
Dillard et al. (2012)b			USA	0.00	144	English	SR	Community	1.931	3.640	0.387	6
Dillard et al. (2012)a			USA	100.00	307	English	SR	Community	2.270	4.190	0.396	6
Feilhauer et al. (2012)	11.670	2.760	Belgium	100.00	76	Dutch	PR	Mixed	2.314	3.578	0.630	6
Stellwagen & Kerig (2012)	12.370	1.750	USA	62.00	100	English	Psychiatrist	Clinical	2.000	5.460	0.690	6
Caldwell et al. (2012)	16.100	0.890	USA	100.00	77	English	SR	Detained			0.670	6
Neal & Sellbom (2012)	19.900	3.480	USA	30.00	602	English	SR	Community	1.590	8.460	0.460	6
Brammer & Lee (2012)	7.400	1.900	USA		106	English	Mixed	Mixed			0.670	6
Crapanzano et al. (2011)	11.280	1.820	USA	45.80	284	English	SR	Community			0.590	6
Roose et al. (2011)	16.850	1.420	Belgium	56.00	830	Dutch	SR	Community	2.080	3.550	0.550	6
Fung (2011)	13.100	1.150	China	60.60	66	Chinese	PR	Community			0.640	6
Eremsoy et al. (2011)b	9.560	1.160	Turkey	52.70	336	Turkish	PR	Community			0.220	6
Eremsoy et al. (2011)a	9.560	1.160	Turkey	52.70	336	Turkish	TR	Community			0.570	6
Sylvers et al. (2011)	8.880	0.980	USA			English	PR	Community		2.070	0.460	6
Javdani et al. (2011)	14.30	1.60	USA	46.00	184	English	SR	Mixed	2.10	3.50	0.56	6
Roose et al. (2010)	16.670	1.340	Belgium	56.00	455	Dutch	SR	Community	2.080	3.550	0.550	6
Fung et al. (2010)	13.030	4.450	China	53.00	3,675		PR	Community	2.070	4.850	0.580	6
Barry et al. (2010)	11.900	1.680	USA	52.04	98	English	Mixed	Community	3.050	4.870	0.720	6
Pardini (2010)	15.830	1.300	USA	53.84	156	English	SR	Detained			0.520	6
Michonski & Sharp (2010)	9.600	1.220	UK	47.81	617	English	PR	Community	2.640	3.230	0.520	6
Stellwagen & Kerig (2010)	11.92	3.04	USA	66.00	100	English	Psychiatrist	Mixed	2.00	5.43	0.680	6
Kerig & Stellwagen (2010)			USA	44.00	252	English	TR	Community	2.269	3.004	0.790	6
Crapanzano et al. (2009)	11.280	1.820	USA	45.80	282	English	SR	Community	2.030	7.540	0.600	6
Stellwagen & Kerig (2009)	12.470	2.960	USA	64.36	101	English	Psychiatrist	Community	2.000	5.460	0.660	6
Sadeh et al. (2009)	14.200	1.600	USA	42.40	229	English	Mixed	Mixed	2.300	5.100	0.610	6
Bijttebier & Decoene (2009)c	13.200	2.470	Belgium	45.05	182	English	SR	Community	1.850	3.340	0.360	6
Bijttebier & Decoene (2009)a	13.200	2.470	Belgium	45.05	182	English	PR	Community	2.100	2.790	0.660	6
Bijttebier & Decoene (2009)b	13.200	2.470	Belgium	45.05	182	English	TR	Community	2.420	3.450	0.700	6

Fite et al. (2009)	8.900	1.980	USA	71.00	328	English	CR	Clinical	2.580	6.320	0.700	6
Kimonis et al. (2008)	15.470	1.370	USA	76.00	248	English	SR	Detained			0.710	6
Howard et al. (2008)	15.500	1.200	USA	87.00	723	English	SR	Detained			0.350	6
Fontaine et al. (2008)	9.000	0.280	UK	46.30	4,713	English	TR	Community			0.630	6
Fite et al. (2008)	8.300	2.400	USA	70.00	212	English	CR	Clinical			0.700	6
Anastassiou-Hadjicharalambous & Warden (2008a)	9.110	0.920	UK	4.92	122	English	Mixed	Mixed		5.270	0.760	6
Anastassiou-Hadjicharalambous & Warden (2008b)	9.340	0.950	UK	94.73	95	English	Mixed	Mixed		6.350	0.760	6
Barry et al. (2007)	11.900	1.680	USA	53.00	98	English	Mixed	Community			0.720	6
Pardini et al. (2007)	10.660	0.570	USA	59.20	116	English	Mixed	Community	2.290	6.190	0.710	6
Muñoz & Frick (2007)a	13.380	1.750	USA	51.65	86	English	SR	Community	1.660	2.720	0.500	6
Muñoz & Frick (2007)b	13.380	1.750	USA	51.65	92	English	PR	Community	2.050	2.410	0.740	6
Pardini (2006)	15.810	1.260	USA	57.40	169	English	SR	Detained			0.520	6
Kimonis et al. (2006a)	3.10	0.68	USA	42.86	49	English	Mixed	Community	2.20	5.65	0.54	6
Kempes et al. (2006)	10.350	1.200	Netherlands	100.00	80		Mixed	Mixed			0.610	6
Dadds et al. (2005)a	6.300	1.100	Australia	0.00	398	English	PR	Community	1.580	2.110	0.440	6
Marsee et al. (2005)a	13.160	1.570	USA	43.00	200	English	TR	Community	2.750	4.610	0.740	6
Frick & Dantagnan (2005)	12.360	1.730	USA	57.00	79	English	Mixed	Community			0.760	6
Frick et al. (2005)	12.360	1.730	USA	47.00	98	English	Mixed	Community	3.090	4.920	0.760	6
Kimonis et al. (2004)	12.360	1.730	USA	47.00	98	English	Mixed	Community			0.760	6
Vitacco et al. (2003)	15.810	1.490	USA	82.58	155	English	SR	Detained	2.83	5.44	0.590	6
Frick et al. (2003)c			USA		92	English	SR	Community	1.660	2.720	0.500	6
Frick et al. (2003)a			USA		98	English	PR	Community	2.500	2.700	0.760	6
Frick et al. (2003)b			USA		98	English	TR	Community	2.970	3.660	0.820	6
Lee et al. (2003)	16.740	1.050	Canada	100.00	100		SR	Detained	1.990	4.590	0.480	6
**Narcissism**												
Andershed et al. (2018)	12.120	0.550	Cyprus	48.00	996		SR	Community			0.730	7
Fanti et al. (2018)b	16.990	1.650	Cyprus	51.00	2,447		SR	Community			0.720	7
Fanti et al. (2018)a	10.250	1.480	Cyprus	48.50	1,471		PR	Community			0.750	7
Romero et al. (2018)	7.320	2.690	Spain	48.60	449	Spanish	TR	Community			0.870	7
Gao et al. (2018)a	11.350	2.610	China	63.50	301	Chinese	PR	Community			0.680	7
Gao et al. (2018)b	11.350	2.610	China	63.50	301	Chinese	SR	Community			0.700	7
Bell et al. (2017)			USA	74.00	77	English	SR	Detained	2.920	4.970	0.730	7
Maurer et al. (2017)	17.380	0.860	USA	100.00	97	English	SR	Detained	2.620	4.390	0.720	7
Leung & Shek (2017)	18.340	1.220	China	43.00	642	Chinese	SR	Community			0.650	7
Lee-Rowland et al. (2017)	16.920	0.840	USA	84.07	358	English	SR	Community	2.470	4.090	0.650	7
Thomson & Centifant (2017)	9.900	0.710	UK	54.55	110	English	TR	Clinical	2.87	4.02	0.870	7
Colins et al. (2017)	16.250	1.070	Belgium	0.00	95		SR	Detained	2.620	4.400	0.660	7
Pechorro et al. (2017f)	16.230	1.380	Portugal	0.00	377	Portuguese	SR	Mixed			0.720	7
Fagan et al. (2017)	9.060	0.600	USA	48.20	340	English	PR	Community	2.006	1.871	0.700	7
Ooi et al. (2017)b	10.60	1.91	Singapore	87.60	282		PR	Clinical	2.770	4.590	0.75	7
Ooi et al. (2017)a	10.60	1.91	Singapore	87.60	282		SR	Clinical	3.280	4.040	0.79	7

Zhang et al. (2017)	8.600	1.650	China	65.09	579	Chinese	PR	Clinical			0.660	7
Robinson et al. (2016)	8.800	0.970	USA	100.00	75	English	Mother	Community	3.250	5.430	0.750	7
Shaffer et al. (2016)	13.070	0.390	Canada	44.48	335		SR	Community	2.060	2.580	0.630	7
Pechorro et al. (2016a)	17.150	1.760	Portugal	15.75	438	Portuguese	SR	Detained			0.760	7
Kahn et al. (2016)	17.030	1.100	USA	83.70	141	English	SR	Detained	2.420	4.490	0.670	7
Rosan et al. (2015)			Romania	100.00	97		SR	Detained	3.080	4.870	0.620	7
Gill & Stickle (2015)a	15.200	1.400	UK	60.00	150	English	SR	Detained			0.640	7
Gill & Stickle (2015)b	15.200	1.400	UK	60.00	150	English	Staff	Detained			0.860	7
Gill & Stickle (2015)c	15.200	1.400	UK	60.00	150	English	TR	Detained			0.790	7
Gill & Stickle (2015)d	15.200	1.400	UK	60.00	150	English	Mixed	Detained			0.570	7
Barry & Lee-Rowland (2015)	16.7	0.72	USA	86.47	211	English	SR	Community	4.240		0.670	7
Pechorro et al. (2015c)	16.750	1.410	Portugal	100.00	221	Portuguese	SR	Detained			0.740	7
Ang et al. (2015)	14.100	1.150	Singapore	58.20	1,027		SR	Community			0.680	7
Oshukova et al. (2015)	15.060	0.280	Finland	46.80	372	Finnish	SR	Community	0.360	0.380	0.760	7
Goodwin et al. (2015)	15.160	1.610	USA	68.07	451	English	SR	Community			0.690	8
Ficks et al. (2014)b	10.570	3.180	USA	0.00	903	English	PR	Community	3.550	2.650	0.670	7
Ficks et al. (2014)a	10.580	3.200	USA	100.00	867	English	PR	Community	3.590	3.080	0.710	7
McKenzie & Lee (2014)a	7.860	1.150	USA		221	English	PR	Mixed			0.800	7
McKenzie & Lee (2014)b	7.860	1.150	USA		153	English	TR	Mixed			0.810	7
Wied et al. (2014)	16.220	0.520	Netherlands	49.00	307	English	Mixed	Community			0.660	7
Fanti et al. (2013)	16.000	0.890	Cyprus	50.30	2,306		SR	Community	3.400	4.130	0.730	7
Dong et al. (2013)b	10.820	3.390	USA	70.00	251		PR	Community	5.560	6.110	0.830	7
Dong et al. (2013)a	10.570	3.190	USA	49.00	1,681		PR	Clinical	3.570	2.880	0.840	7
Muñoz et al. (2013)	15.500	1.280	USA	100.00	85	English	SR	Detained	2.830	4.210	0.710	7
Kauten et al. (2013)	17.050	0.880	USA	83.77	154	English	SR	Community	2.290	4.400	0.600	7
Dillard et al. (2012)b			USA	0.00	144	English	SR	Community	2.635	4.380	0.619	7
Dillard et al. (2012)a			USA	100.00	307	English	SR	Community	3.055	4.340	0.771	7
Feilhauer et al. (2012)	11.670	2.760	Belgium	100.00	76	Dutch	PR	Mixed	3.419	5.220	0.800	7
Stellwagen & Kerig (2012)	12.370	1.750	USA	62.00	100	English	Psychiatrist	Clinical	3.180	5.810	0.790	7
Sellbom & Phillips (2012)b	19.050	1.730	USA	32.54	627	English	SR	Mixed			0.690	7
Caldwell et al. (2012)	16.100	0.890	USA	100.00	77	English	SR	Detained			0.690	7
Neal & Sellbom (2012)	19.900	3.480	USA	30.00	602	English	SR	Community	2.260	10.470	0.670	7
Roose et al. (2011)	16.850	1.420	Belgium	56.00	830	Dutch	SR	Community	2.580	4.290	0.670	7
Fung (2011)	13.100	1.150	China	60.60	66	Chinese	PR	Community			0.760	7
Sylvers et al. (2011)	8.880	0.980	USA			English	PR	Community		3.110	0.720	7
Javdani et al. (2011)	14.30	1.60	USA	46.00	184	English	SR	Mixed	2.30	3.80	0.66	7
Roose et al. (2010)	16.670	1.340	Belgium	56.00	455	Dutch	SR	Community	2.580	4.290	0.670	7
Fung et al. (2010)	13.030	4.450	China	53.00	3,675	Chinese	PR	Community	2.750	4.410	0.760	7
Barry & Malkin (2010)	16.720	0.710	USA	80.34	534	English	SR	Community	2.670	4.100	0.700	7
Barry & Wallace (2010)	17.100	0.930	USA	87.18	117	English	SR	Community	2.210	4.250	0.600	7
Barry et al. (2010)	11.900	1.680	USA	52.04	98	English	Mixed	Community	3.620	5.080	0.840	7
Michonski & Sharp (2010)	9.600	1.220	UK	47.81	617	English	PR	Community	1.980	2.370	0.670	7
Hall et al. (2010a)	15.500	1.250	USA	87.00	723	English	SR	Detained			0.750	7

Kerig & Stellwagen (2010)			USA	44.00	252	English	TR	Community	2.990	2.757	0.870	7
Stellwagen & Kerig (2009)	12.470	2.960	USA	64.36	101	English	Psychiatrist	Community			0.780	7
Sadeh et al. (2009)	14.200	1.600	USA	42.40	229	English	Mixed	Mixed	2.800	5.300	0.730	7
Bijttebier & Decoene (2009)c	13.200	2.470	Belgium	45.05	182	English	SR		2.340	3.830	0.620	7
Bijttebier & Decoene (2009)a	13.200	2.470	Belgium	45.05	182	English	PR		2.270	2.570	0.750	7
Bijttebier & Decoene (2009)b	13.200	2.470	Belgium	45.05	182	English	TR		2.510	1.950	0.810	7
Fite et al. (2009)	8.900	1.980	USA	71.00	328	English	CR	Clinical	3.380	7.280	0.760	7
Lee et al. (2009)a	16.11	1.44	Canada	100.00	112	English	SR	Detained	2.76	4.73	0.75	7
Muñoz et al. (2007)	15.530	1.280	USA	100.00	100	English	SR	Detained	2.870	4.020	0.720	7
Howard et al. (2008)	15.500	1.200	USA	87.00	723	English	SR	Detained			0.750	7
Fontaine et al. (2008)	9.000	0.280	UK	46.30	4,713	English	TR	Community			0.830	7
Fite et al. (2008)	8.300	2.400	USA	70.00	212	English	CR	Clinical			0.760	7
Murrieet al. (2007)	15.970	1.550	USA	100.00	490	English	SR	Detained			0.700	7
Muñoz & Frick (2007)a	13.380	1.750	USA	51.65	86	English	SR	Community	2.040	3.150	0.630	7
Muñoz & Frick (2007)b	13.380	1.750	USA	51.65	92	English	PR	Community	2.870	3.220	0.820	7
Dadds et al. (2005)b	6.300	1.100	Australia	100.00	302	English	PR	Community	2.640	3.110	0.780	7
Marsee et al. (2005)a	13.160	1.570	USA	43.00	200	English	TR	Community	2.810	2.030	0.870	7
Spain et al. (2004)	15.770	1.350	USA	100.00	85	English	SR	Detained			0.690	7
Vitacco et al. (2003)	15.810	1.490	USA	82.58	155	English	SR	Detained	2.80	5.00	0.740	7
Frick et al. (2003)c			USA		92	English	SR	Community	2.040	3.150	0.630	7
Frick et al. (2003)a			USA		98	English	PR	Community	2.980	3.230	0.820	7
Frick et al. (2003)b			USA		98	English	TR	Community	3.570	3.280	0.900	7
Lee et al. (2003)	16.740	1.050	Canada	100.00	100		SR	Detained	2.620	5.930	0.660	7
**Impulsivity**												
Andershed et al. (2018)	12.120	0.550	Cyprus	48.00	996		SR	Community			0.700	5
Fanti et al. (2018)b	16.990	1.650	Cyprus	51.00	2,447		SR	Community			0.640	5
Fanti et al. (2018)a	10.250	1.480	Cyprus	48.50	1,471		PR	Community			0.700	5
Romero et al. (2018)	7.320	2.690	Spain	48.60	449	Spanish	TR	Community			0.810	5
Colins et al. (2018)	8.840	1.470	Cyprus	46.52	690	English	PR	Community			0.730	5
Gao et al. (2018)b	11.350	2.610	China	63.50	301	Chinese	SR	Community			0.630	5
Gao et al. (2018)a	11.350	2.610	China	63.50	301	Chinese	PR	Community			0.690	5
Maurer et al. (2017)	17.380	0.860	USA	100.00	97	English	SR	Detained	1.920	5.140	0.610	5
Lee-Rowland et al. (2017)	16.920	0.840	USA	84.07	358	English	SR	Community			0.500	5
Thomson & Centifant (2017)	9.900	0.710	UK	54.55	110	English	TR	Clinical		4.24	0.800	5
Colins et al. (2017)	16.250	1.070	Belgium	0.00	95		SR	Detained	2.180	5.460	0.630	5
Pechorro et al. (2017f)	16.230	1.380	Portugal	0.00	377	Portuguese	SR	Mixed			0.560	5
Fagan et al. (2017)	9.060	0.600	USA	48.20	340	English	PR	Community	1.753	2.604	0.600	5
Ooi et al. (2017)b	10.60	1.91	Singapore	87.60	282		PR	Clinical	1.650	5.730	0.49	5
Ooi et al. (2017)a	10.60	1.91	Singapore	87.60	282		SR	Clinical	2.430	4.150	0.69	5
Zhang et al. (2017)	8.600	1.650	China	65.09	579	Chinese	PR	Clinical			0.650	5
Robinson et al. (2016)	8.800	0.970	USA	100.00	75	English	Mother	Community	2.000	6.150	0.63	5
Shaffer et al. (2016)	13.070	0.390	Canada	44.48	335		SR	Community	1.730	3.320	0.510	5
Pechorro et al. (2016a)	17.150	1.760	Portugal	15.75	438	Portuguese	SR	Detained			0.600	5

Rosan et al. (2015)			Romania	100.00	97		SR	Detained	2.240	4.080	0.600	5
Gill & Stickle (2015)c	15.200	1.400	UK	60.00	150	English	TR	Detained			0.70	5
Gill & Stickle (2015)a	15.200	1.400	UK	60.00	150	English	SR	Detained			0.72	5
Gill & Stickle (2015)d	15.200	1.400	UK	60.00	150	English	Mixed	Detained			0.73	5
Gill & Stickle (2015)b	15.200	1.400	UK	60.00	150	English	Staff	Detained			0.77	5
Williford et al. (2015)	16.050	1.300	USA	45.00	137	English	SR	Community			0.620	5
Goodwin et al. (2015)	15.160	1.610	USA	68.07	451	English	SR	Community			0.580	5
Sellbom et al. (2015)	19.120	1.430	USA	40.29	278	English	SR	Mixed			0.550	5
Pechorro et al. (2015c)	16.750	1.410	Portugal	100.00	221	Portuguese	SR	Detained			0.600	5
Oshukova et al. (2015)	15.060	0.280	Finland	46.80	372	Finnish	SR	Community	0.430	0.700	0.680	5
Sharp & Vanwoerden (2014)	15.390	1.450	USA	38.50	346	English	SR	Clinical	1.900	5.630	0.560	5
Ficks et al. (2014)a	10.580	3.200	USA	100.00	867	English	PR	Community	3.670	4.410	0.780	5
Ficks et al. (2014)b	10.570	3.180	USA	0.00	903	English	PR	Community	3.220	3.450	0.780	5
Wied et al. (2014)	16.220	0.520	Netherlands	49.00	307	English	Mixed	Community			0.300	5
Fanti et al. (2013)	16.000	0.890	Cyprus	50.30	2,306		SR	Community	2.850	4.870	0.690	5
Dong et al. (2013)a	10.570	3.190	USA	49.00	1,681		PR	Clinical	3.480	3.910	0.780	5
Dong et al. (2013)b	10.820	3.390	USA	70.00	251		PR	Community	4.500	9.560	0.720	5
Muñoz et al. (2013)	15.500	1.280	USA	100.00	85	English	SR	Detained	2.030	4.530	0.570	5
Kahn et al. (2013)a	13.430	1.860	USA	51.00	272	English	SR	Clinical	1.95	4.62	0.600	5
Kahn et al. (2013)b	13.430	1.860	USA	51.00	272	English	PR	Clinical	2.32	5.72	0.700	5
Dillard et al. (2012)b			USA	0.00	144	English	SR	Community	2.119	4.750	0.554	5
Dillard et al. (2012)a			USA	100.00	307	English	SR	Community	2.046	4.410	0.630	5
Feilhauer et al. (2012)	11.670	2.760	Belgium	100.00	76	Dutch	PR	Mixed	2.589	4.560	0.790	5
Stellwagen & Kerig (2012)	12.370	1.750	USA	62.00	100	English	Psychiatrist	Clinical	2.060	6.120	0.710	5
Sellbom & Phillips (2012)b	19.050	1.730	USA	32.54	627	English	SR	Mixed			0.590	5
Caldwell et al. (2012)	16.100	0.890	USA	100.00	77	English	SR	Detained			0.710	5
Neal & Sellbom (2012)	19.900	3.480	USA	30.00	602	English	SR	Community	1.810	8.640	0.540	5
Roose et al. (2011)	16.850	1.420	Belgium	56.00	830	Dutch	SR	Community	2.150	4.340	0.620	5
Fung (2011)	13.100	1.150	China	60.60	66	Chinese	PR	Community			0.580	5
Sylvers et al. (2011)	8.880	0.980	USA			English	PR	Community		1.830	0.580	5
Javdani et al. (2011)	14.30	1.60	USA	46.00	184	English	SR	Mixed	1.90	4.20	0.53	5
Roose et al. (2010)	16.670	1.340	Belgium	56.00	455	Dutch	SR	Community	2.150	4.340	0.620	5
Fung et al. (2010)	13.030	4.450	China	53.00	3,675		PR	Community	2.050	3.270	0.640	5
Michonski & Sharp (2010)	9.600	1.220	UK	47.81	617	English	PR	Community	1.820	3.900	0.640	5
Hall et al. (2010b)	15.800	1.100	USA	83.80	247	English	SR	Clinical	1.560	2.030	0.750	5
Hall et al. (2010a)	15.500	1.250	USA	87.00	723	English	SR	Detained	2.170	6.460	0.670	5
Kerig & Stellwagen (2010)			USA	44.00	252	English	TR	Community	2.227	2.976	0.790	5
Crapanzano et al. (2009)	11.280	1.820	USA	45.80	282	English	SR	Community	1.610	6.720	0.510	5
Stellwagen & Kerig (2009)	12.470	2.960	USA	64.36	101	English	Psychiatrist	Community			0.660	5
Sadeh et al. (2009)	14.200	1.600	USA	42.40	229	English	Mixed	Mixed	2.000	5.600	0.650	5
Bijttebier & Decoene (2009)a	13.200	2.470	Belgium	45.05	182	English	PR		1.900	2.900	0.590	5
Fite et al. (2009)	8.900	1.980	USA	71.00	328	English	CR	Clinical	2.300	6.510	0.670	5
Lee et al. (2009)a	16.11	1.44	Canada	100.00	112	English	SR	Detained	1.91	5.34	0.58	5

Greening et al. (2008)	9.810	1.330	USA	100.00	87	English	PR	Clinical			0.730	5
Muñoz et al. (2007)	15.530	1.280	USA	100.00	100	English	SR	Detained	2.170	4.240	0.600	5
Howard et al. (2008)	15.500	1.200	USA	87.00	723	English	SR	Detained			0.670	5
Fontaine et al. (2008)	9.000	0.280	UK	46.30	4,713	English	TR	Community			0.740	5
Fite et al. (2008)	8.300	2.400	USA	70.00	212	English	CR	Clinical			0.670	5
Murrieet al. (2007)	15.970	1.550	USA	100.00	490	English	SR	Detained			0.620	5
Muñoz & Frick (2007)a	13.380	1.750	USA	51.65	86	English	SR	Community	1.940	4.040	0.680	5
Muñoz & Frick (2007)b	13.380	1.750	USA	51.65	92	English	PR	Community	2.100	4.080	0.730	5
Marsee et al. (2005)a	13.160	1.570	USA	43.00	200	English	TR	Community	2.170	2.340	0.710	
Spain et al. (2004)	15.770	1.350	USA	100.00	85	English	SR	Detained			0.560	
Vitacco et al. (2003)	15.810	1.490	USA	82.58	155	English	SR	Detained	1.90	4.91	0.530	
Frick et al. (2003)c			USA		92	English	SR	Community	2.010	4.130	0.680	5
Frick et al. (2003)a			USA		98	English	PR	Community	2.060	4.030	0.720	5
Frick et al. (2003)b			USA		98	English	TR	Community	2.830	3.660	0.830	5
Lee et al. (2003)	16.740	1.050	Canada	100.00	100		SR	Detained	1.870	5.860	0.570	5
**Total**												
Jones et al. (2009)	9.000		UK	52.41	1,284	English	PR	Community			0.75	20
Roose et al. (2011)	16.850	1.420	Belgium	56.00	830	Dutch	SR	Community	6.140	13.520	0.820	20
Roose et al. (2010)	16.670	1.340	Belgium	56.00	455	Dutch	SR	Community	6.140	13.520	0.820	20
Brouns et al. (2013)b	16.680	0.410	Netherlands	46.00	233	Dutch	PR	Community	4.260	8.430	0.760	20
Brouns et al. (2013)a	16.680	0.410	Netherlands	46.00	233	Dutch	PR	Community	4.070	7.190	0.770	20
Goodwin et al. (2015)	15.160	1.610	USA	68.07	451	English	SR	Community			0.800	20
Eremsoy et al. (2011)b	9.560	1.160	Turkey	52.70	336	Turkish	PR	Community			0.730	20
Eremsoy et al. (2011)a	9.560	1.160	Turkey	52.70	336	Turkish	TR	Community			0.860	20
Shaffer et al. (2016)	13.070	0.390	Canada	44.48	335		SR	Community	4.350	9.440	0.730	20
Tuvblad et al. (2014)b	14.870	0.870	USA		1,010	English	PR	Community			0.840	20
Tuvblad et al. (2014)a	14.870	0.870	USA		1,076	English	SR	Community			0.740	20
Gillen et al. (2019)	15.930	1.290	USA	85.00	60	English	SR	Detained	5.250	13.680	0.880	20
Dillard et al. (2012)b			USA	0.00	144	English	SR	Community	5.142	13.280	0.720	20
Dillard et al. (2012)b			USA	100.00	307	English	SR	Community	5.986	13.280	0.801	20
Murrieet al. (2007)	15.970	1.550	USA	100.00	490	English	SR	Detained			0.780	20
Murrie et al. (2004)a	16.000	1.100	USA	100.00	113	English	Mixed	Detained			0.880	20
Murrie et al. (2004)b	16.000	1.100	USA	100.00	113	English	SR	Detained			0.710	20
Garland et al. (2011)	15.490	1.240	USA	87.02	723	English	SR	Detained	5.350	16.260	0.700	20
Garland & Howard (2010)	15.460	1.220	USA	86.99	723	English	SR	Detained	5.550	16.230	0.700	20
Garland et al. (2009)	15.500	1.200	USA	87.00	723	English	SR	Community	5.410	16.710	0.700	20
Kimonis et al. (2006b)	9.300	2.000	USA	54.00	50	English	SR	Community	5.250	14.780	0.790	20
Lindblad et al. (2015)	16.400	0.900	Russia	100.00	370	Russian	Mixed	Detained			0.790	20
Bell et al. (2017)			USA	74.00	77	English	SR	Detained			0.730	20
Wendt et al. (2017)			UK	45.10	249	English	SR	Community			0.710	20
Maurer et al. (2017)	17.380	0.860	USA	100.00	97	English	SR	Detained	5.800	16.140	0.680	20
Goodnight et al. (2017)	11.610	7.480	USA	48.50	446	English	SR	Community	4.960	10.750	0.730	20
Anderson et al. (2012)	19.900	3.880	USA	28.90	384	English	SR	Mixed			0.850	20

Michonski & Sharp (2010)	9.600	1.220	UK	47.81	617	English	PR	Community	4.610	9.030	0.810	20
Vitale et al. (2005)b	12.000	4.220	USA	0.00	144	English	SR	Community			0.740	20
Vitale et al. (2005)a	16.390	3.280	USA	100.00	164	English	SR	Community			0.770	20
Feilhauer et al. (2012)	11.670	2.760	Belgium	100.00	76	Dutch	PR	Mixed	7.246	13.365	0.880	20
Edens et al. (2011)	15.490	1.230	USA	87.00	722	English	SR	Detained			0.830	20
Racer et al. (2011)	11.760	1.330	USA	40.74	54	English	PR	Community			0.830	20
Drislane et al. (2015)	18.800	1.630	USA	44.40	618	English	SR	Community			0.780	20
Romero et al. (2018)	7.320	2.690	Spain	48.60	449	Spanish	TR	Community			0.910	20
Barrutieta et al. (2015)	17.700	1.160	Spain	100.00	129	Spanish	TR	Community			0.974	20
Muñoz & Frick (2007)b	13.380	1.750	USA	51.65	92	English	PR	Community	6.320	10.370	0.880	20
Muñoz & Frick (2007)a	13.380	1.750	USA	51.65	86	English	SR	Community	5.140	10.710	0.810	20
Fritz et al. (2008)	16.400	0.900	Russia	100.00	175	Russian	SR	Detained	6.070	17.050	0.790	20
Sellbom et al. (2015)	19.120	1.430	USA	40.29	278	English	SR	Mixed			0.780	20
Caldwell et al. (2012)	16.100	0.890	USA	100.00	77	English	SR	Detained			0.780	20
Vaughn et al. (2008a)	15.400	1.100	USA	0.00	94	English	SR	Detained			0.850	20
Vaughn et al. (2009)			USA		723	English	SR	Detained			0.830	20
Wied et al. (2014)	16.220	0.520	Netherlands	49.00	307	English	Mixed	Community			0.730	20
Vitacco et al. (2003)	15.810	1.490	USA	82.58	155	English	SR	Detained	6.360	17.490	0.620	20
Sobhan et al. (2015)	18.900	0.700	USA	100.00	24	English	SR	Community			0.740	20
Marsee et al. (2005)b	13.160	1.570	USA	43.00	200	English	SR	Community	4.930	13.320	0.710	20
Marsee et al. (2005)a	13.160	1.570	USA	43.00	200	English	TR	Community	7.130	9.330	0.900	20
Sadeh et al. (2009)	14.200	1.600	USA	42.40	229	English	Mixed	Mixed	6.400	17.400	0.840	20
Thomson & Centifant (2017)	9.900	0.710	UK	54.55	110	English	TR	Clinical			0.910	20
Colins et al. (2017)	16.250	1.070	Belgium	0.00	95		SR	Detained	5.400	15.870	0.730	20
Bijttebier & Decoene (2009)a	13.200	2.470	Belgium	45.05	182	English	PR		5.450	8.600	0.840	20
Sylvers et al. (2011)	8.880	0.980	USA		88	English	PR	Community		5.600	0.800	20
Fite et al. (2009)	8.900	1.980	USA	71.00	328	English	Mixed	Clinical	7.260	21.770	0.850	20
Frick et al. (2003)a			USA		98	English	PR	Community	6.470	10.480	0.890	20
Frick et al. (2003)c			USA		92	English	SR	Community	5.140	10.710	0.810	20
Frick et al. (2003)b			USA		98	English	TR	Community	8.510	11.140	0.920	20
Fite et al. (2008)	8.300	2.400	USA	70.00	212	English	Mixed	Clinical			0.850	20
Pechorro et al. (2014b)	16.010	1.170	Portugal	100.00	160	Portuguese	SR	Detained	5.300	14.820	0.710	20
Pechorro et al. (2015c)	16.750	1.410	Portugal	100.00	221	Portuguese	SR	Detained			0.810	20
Pechorro et al. (2017h)	16.750	1.410	Portugal	100.00	221	Portuguese	SR	Detained	6.660	20.550	0.810	20
Pechorro et al. (2017f)	16.230	1.380	Portugal	0.00	377	Portuguese	SR	Mixed			0.770	20
Pechorro et al. (2016f)	16.750	1.410	Portugal	100.00	221	Portuguese	SR	Detained			0.810	20
Pechorro et al. (2015a)	16.750	1.410	Portugal	0.00	21	Portuguese	SR	Detained			0.810	20
Pechorro et al. (2017a)	16.230	1.380	Portugal	0.00	377	Portuguese	SR	Mixed			0.770	20
Pechorro et al. (2014d)	15.900	1.350	Portugal	100.00	306	Portuguese	SR	Mixed			0.750	20
Pechorro et al. (2017e)c	16.170	1.440	Portugal	0.00	274	Portuguese	SR	Community			0.740	20
Pechorro et al. (2017e)b	16.410	1.190	Portugal	0.00	103	Portuguese	SR	Detained			0.720	20
Pechorro et al. (2017e)a	16.230	1.380	Portugal	0.00	377	Portuguese	SR	Mixed			0.810	20
Pechorro et al. (2016e)	15.870	1.720	Portugal	47.44	782	Portuguese	SR	Community			0.770	20

Pechorro et al. (2016d)	15.870	1.720	Portugal	47.44	782	Portuguese	SR	Community			0.770	20
Pechorro et al. (2016c)	16.640	1.360	Portugal	68.20	324	Portuguese	SR	Detained			0.740	20
Pechorro et al. (2017g)	15.870	1.720	Portugal	47.44	782	Portuguese	SR	Community			0.770	20
Pechorro et al. (2017c)	16.230	1.380	Portugal	0.00	337	Portuguese	SR	Mixed			0.770	20
Pechorro et al. (2014a)	15.690	1.220	Portugal	0.00	132	Portuguese	SR	Mixed			0.820	20
Pechorro et al. (2012)	15.960	1.490	Portugal	100.00	543	Portuguese	SR	Mixed			0.750	20
Pechorro et al. (2017d)	16.750	1.410	Portugal	100.00	221	Portuguese	SR	Detained			0.810	20
Pechorro et al. (2016b)	16.230	1.380	Portugal	0.00	377	Portuguese	SR	Mixed			0.770	20
Pechorro et al. (2015b)a	15.830	1.300	Portugal	100.00	201		SR	Detained	4.956	15.565	0.700	20
Pechorro et al. (2015b)b	15.960	1.280	Portugal	0.00	98		SR	Detained	5.702	15.297	0.750	20
Pechorro et al. (2016a)	17.150	1.760	Portugal	15.75	438	Portuguese	SR	Detained			0.830	20
Pechorro et al. (2014c)	15.810	1.230	Portugal	0.00	236	Portuguese	SR	Mixed	5.390	10.530	0.770	20
Muratori et al. (2017)	14.360	1.310	Italy	90.91	55	Italian	PR	Detained			0.770	20
Kahn et al. (2016)	17.030	1.100	USA	83.70	141	English	SR	Detained	4.240	14.820	0.750	20
Ang et al. (2015)	14.100	1.150	Singapore	58.20	1,027		SR	Community			0.710	20
Javdani et al. (2011)	14.300	1.600	USA	46.00	184	English	SR	Mixed	5.200	12.500	0.740	20
Snyder et al. (2015)	15.500	1.200	USA	87.00	722	English	SR	Community	5.500	16.300	0.700	20
Oshukova et al. (2015)	15.060	0.280	Finland	46.80	372	Finnish	SR	Community	0.790	1.710	0.790	20
Laajasalo et al. (2014)	15.300	0.550	Finland	49.00	4,855		SR	Community			0.760	20
Neal & Sellbom (2012)	19.900	3.480	USA	30.00	602	English	SR	Community	4.830	30.770	0.760	20
Ooi et al. (2017)b	10.600	1.910	Singapore	87.60	282		PR	Clinical	4.950	17.490	0.760	20
Ooi et al. (2017)a	10.600	1.910	Singapore	87.60	282		SR	Clinical	5.840	15.010	0.730	20
Lee et al. (2003)	16.740	1.050	Canada	100.00	100		SR	Detained	5.420	18.660	0.770	20
Lee et al. (2009)a	16.110	1.440	Canada	100.00	112	English	SR	Detained	5.490	16.740	0.750	20
Liu et al. (2016)b	15.500	0.670	China	50.80	368	Chinese	SR	Community	5.370	34.260	0.720	20
Liu et al. (2016)a	15.000	1.580	China	58.60	1,067	Chinese	SR	Community	4.320	26.990	0.780	20
Zhang et al. (2017)	8.600	1.650	China	65.09	579	Chinese	PR	Clinical			0.810	20

Notes. PR = Parent-Report; SR = Self-Report; TR = Teacher-Report; CR = Caregiver-Report; α = Cronbach’s alpha coefficient; N = sample size; M = Mean; SD = Standard deviation.
